# Surgery and Medical Treatment in Microprolactinoma: A Systematic Review and Meta-Analysis

**DOI:** 10.1155/2021/9930059

**Published:** 2021-08-30

**Authors:** Jianglong Lu, Lin Cai, Zerui Wu, Weiwei Lin, Jiadong Xu, Zhangzhang Zhu, Chengde Wang, Qun Li, Zhipeng Su

**Affiliations:** ^1^Department of Neurosurgery, First Affiliated Hospital of Wenzhou Medical University, Wenzhou 325000, China; ^2^Department of Neurosurgery, Wencheng Country People's Hospital, Wenzhou 325000, China

## Abstract

**Objective:**

Dopamine agonists (DAs) are recommended as the first-line treatment for prolactinomas; however, tumour recurrence after drug withdrawal remains a clinical problem. Recent studies have reported high remission rates via surgery in microprolactinomas. The aim of this systematic review and meta-analysis was to compare the clinical result of DA treatment with surgery as initial therapy in patients with treatment-naive microprolactinoma.

**Methods:**

A comprehensive literature search for studies and reports regarding microprolactinoma patients treated with DAs and/or surgery published between January 1970 and November 2020 was conducted using four electronic databases (PubMed, Embase, Google Scholar, and the Cochrane Library). Clinical treatment outcome was evaluated by the biochemical remission of serum prolactin level to normal after treatment. The *I*^2^ statistic was used to quantify heterogeneity. Pooled data were analysed according to a random effect model.

**Results:**

Eighteen studies with 661 patients were included for analysis. The DA treatment group achieved a higher remission rate at ≥12 months follow-up (96% vs. 86%; *P*=0.019). Surgery showed a higher remission rate than the DA treatment group after the treatment withdrawal (78% vs. 44%; *P*=0.003). Patients with preoperative prolactin level of ≤200 ng/mL had a higher remission rate than patients with preoperative prolactin level of >200 ng/mL (92% vs. 40%; *P*=0.029).

**Conclusion:**

Surgery showed a high remission rate in treatment-naive microprolactinoma patients after treatment withdrawal and may be an alternative first-line treatment strategy in addition to DAs, particularly in patients with a preoperative prolactin level of ≤200 ng/mL.

## 1. Introduction

As the most common subtype of pituitary adenomas, prolactinomas account for approximately 32%–66% of all pituitary adenomas [1]. More than 90% are microprolactinomas (size: <10 mm) [2, 3]. Infertility and gonadal and sexual dysfunction are the most relevant clinical features in both women and men [4, 5]. Therapeutic options include pharmacotherapy, surgery, and radiation. Since the mid-1980s, surgery and radiotherapy have been progressively replaced by pharmacotherapy with dopamine agonists (DAs) [6]. These agents result in prolactin (PRL) normalisation in approximately 75%–90% of prolactinoma cases [7] and are therefore recommended as first-line therapy [1, 8]. However, tumour recurrence after withdrawal of DA therapy remains a clinical problem. Recurrence of hyperprolactinemia after DA withdrawal reportedly ranges from 33.9% to 100%, even if the PRL had normalised during DA treatment for >2 years [9, 10].

Surgery as another therapeutic option has been applied for more than 100 years. Some studies have reported normalisation of PRL after surgery for macroprolactinoma in only 30%–45% of patients [4, 11, 12], which is considerably lower than medical treatment. However, recent studies have reported high PRL normalisation rates after surgery for microprolactinomas [13–18] that were near or superior to DA treatment. Andereggen et al. [15] reported that 37 of 41 microprolactinoma patients (90.2%) maintained a normal PRL level at 90 months of median follow-up after primary surgical therapy and recommended that a primary surgical approach should be interdisciplinarily discussed in these patients. Gnjidic et al. [19] reported early normalisation of PRL in 98% of microprolactinoma patients who underwent primary surgical treatment. Therefore, some clinical experts have suggested that surgery may be an alternative treatment option for patients with microprolactinoma [20, 21]. To determine the optimal treatment strategy, we conducted this systematic review and meta-analysis to compare the efficacy of DAs and surgery as the initial therapy strategy in treatment-naive microprolactinoma patients.

## 2. Methods

The current systematic review was written and followed according to the preferred reporting items for systematic reviews and meta-analyses statement guidelines [22, 23].

### 2.1. Study Eligibility

The following study criteria were used to determine eligibility for inclusion in this meta-analysis:

1. Participants: Microprolactinomas (diameter: <10 mm) must be diagnosed clearly. Neither age nor gender was restricted.
2. Interventions: Patients received either medical treatment or surgical treatment as initial therapy. We restricted medical treatment to the DAs, which was either bromocriptine (BRC) and/or cabergoline (CAB). Surgical treatment consisted of transsphenoidal surgery (TSS) for tumour removal using a microscope or endoscope. Those who received surgery and/or radiation before DAs were eliminated from the medical treatment group; if a study did not specify whether the patients received either treatment prior to therapy with DAs, the concerned study was also excluded. If a study did not specify receipt and/or discontinuation of DA therapy prior to surgery, the study was excluded. The studies must contain clear and definite data about normalisation of PRL after treatment. Patient clinical characteristics including age, gender, mean follow-up time, and the name of DAs should be clearly described.
3. Outcome measures: The accurate remission rate must be provided directly or can be calculated from the original data.
4. Study types: We collected all types of studies except clinical reviews; however, the case reports should include at least three patients.

### 2.2. Literature Search

We conducted a wide search from databases including PubMed, Embase, Google Scholar, and the Cochrane Library to extract all the studies on treatments using a surgical or medical approach for patients with microprolactinoma published between January 1970 and November 2020 (as shown in Supplemental Table 1). Two reviewers independently scanned abstracts and headlines to distinguish if they were eligible for further review. For all potential articles, the manuscript or full-text version was available for the reviewers to check thoroughly again. Controversial potential articles were discussed and eventually a consensus between the two reviewers was reached. Furthermore, we also conducted a manual search of related reference lists to expand the search. There was no language restriction.

### 2.3. Data Extraction

Two reviewers (JiangLong Lu and Lin Cai) independently screened information regarding author, year of publication, patient demographics and baseline characteristics, sample size, risk factors, PRL levels, remission rate, follow-up period, time of remission, and microprolactinoma-related characteristics. If a study including all sizes of adenomas, we extract the data of microadenomas only from original studies or contact the author to get the original data.

### 2.4. Heterogeneity and Risk of Bias Assessment

We utilized the *I*^2^ statistic to evaluate the heterogeneity, which describes the percentage of variation between studies caused by heterogeneity rather than chance [24]. If heterogeneity was high (*I*^2^ > 50%; *P* > 0.1), the DerSimonian and Laird random-effects model was used for the summary statistics [25]. Besides, sensitivity analysis was further investigated to detect potential sources of heterogeneity.

For the included studies, a modified version of the Newcastle–Ottawa quality assessment scale [26] for cohort studies was adopted to assess the quality and risk of bias. In addition, the risk of bias in the subgroup analysis was evaluated using funnel plots and Egger's test.

### 2.5. Outcome

The primary outcome of interest was the biochemical remission rate of microprolactinoma. Biochemical remission was strictly defined as the return to normal serum PRL level after treatment.

### 2.6. Statistical Analysis and Data Synthesis

Cumulative rate estimates over time were computed with the variance-stabilising double-arcsine transformation [27]. We utilized the accurate method [28] to calculate a 95% confidence interval (CI) based on these estimates because the asymptotic method can generate intervals that may extend below zero [29]. The outcomes between medical and surgical treatment were compared using the Student's *t*-test. Subgroup analyses were conducted based on the follow-up period and the preoperative PRL level.

Statistical analysis was performed using STATA software version 14.2 (StataCorp LP, College Station, TX, USA) with the commands “metaprop” specifying three variables: double-arcsine-transformed prevalence, exact CIs, and remission rates in a random-effects model; “metaninf”; and “metabias.”

## 3. Results

**Figure 1:**
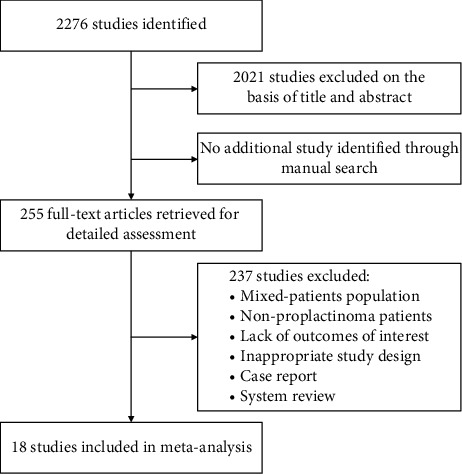
Flowchart of the literature search yield and selected studies.

Figure 1 shows the flowchart for our literature search. Our initial search strategy found a total of 2,276 articles. After initial screening and excluding duplicates, 255 papers remained for further review. The final sample included 18 studies (661 patients) published between 1999 and 2018 after the exclusion of ineligible reports. These studies included 16 case series and 2 retrospective cohort studies. Eight studies included patients who accepted DA treatment initially; 8 studies included patients who accepted surgical treatment initially; and the remaining 2 studies included both medical and surgical treatment patients. The demographic characteristics of the included studies were presented in Table 1. Mean patient follow-up was >5 years in 5 studies; mean follow-up in the remaining studies ranged from 2 to 4 years. Therefore, we conducted a prespecified sensitivity analysis. The results of the modified Newcastle–Ottawa quality assessment for the articles included in this meta-analysis are shown in Supplemental Table 2.

### 3.1. Meta-Analysis

**Figure 2:**
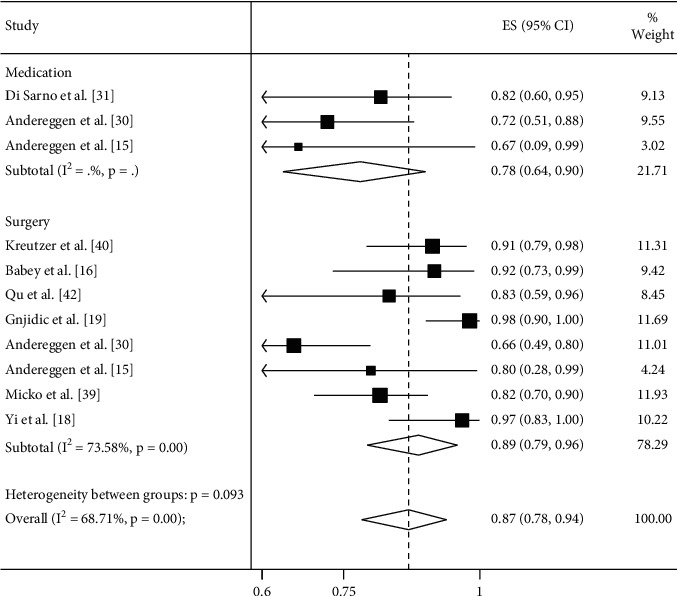
Meta-analysis of remission rates comparing surgery and medical treatment in microprolactinomas at ≤3 months of follow-up. The remission rate was achieved in 89% of patients treated with surgery and 78% of patients treated with DAs (*P*=0.092) at ≤3 months of follow-up.

**Figure 3:**
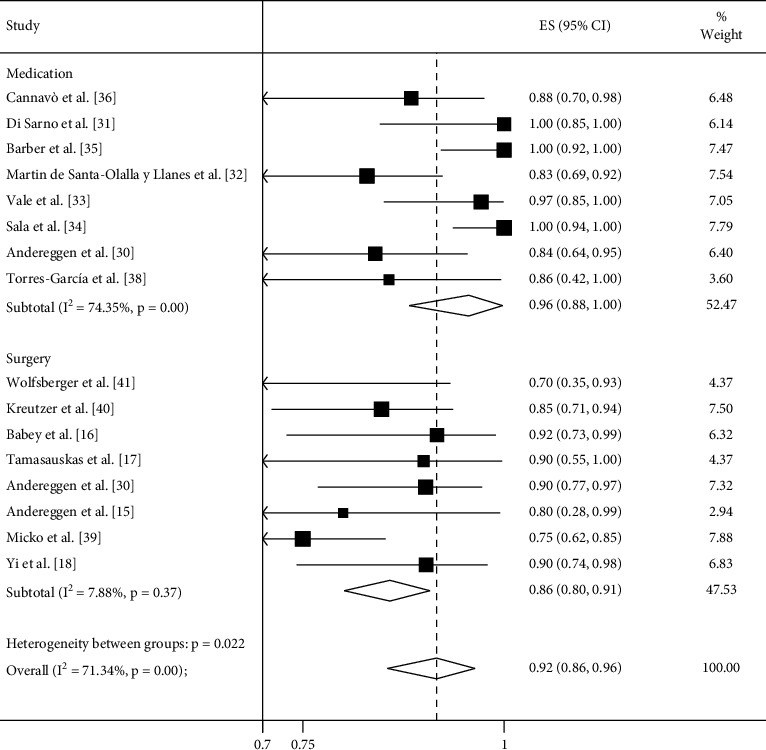
Meta-analysis of remission rates comparing surgery and medical treatment in microprolactinomas at ≥12 months of follow-up. The remission rate was achieved in 86% of patients treated with surgery and 96% of patients treated with DAs (*P*=0.019) at ≥12 months of follow-up.

**Figure 4:**
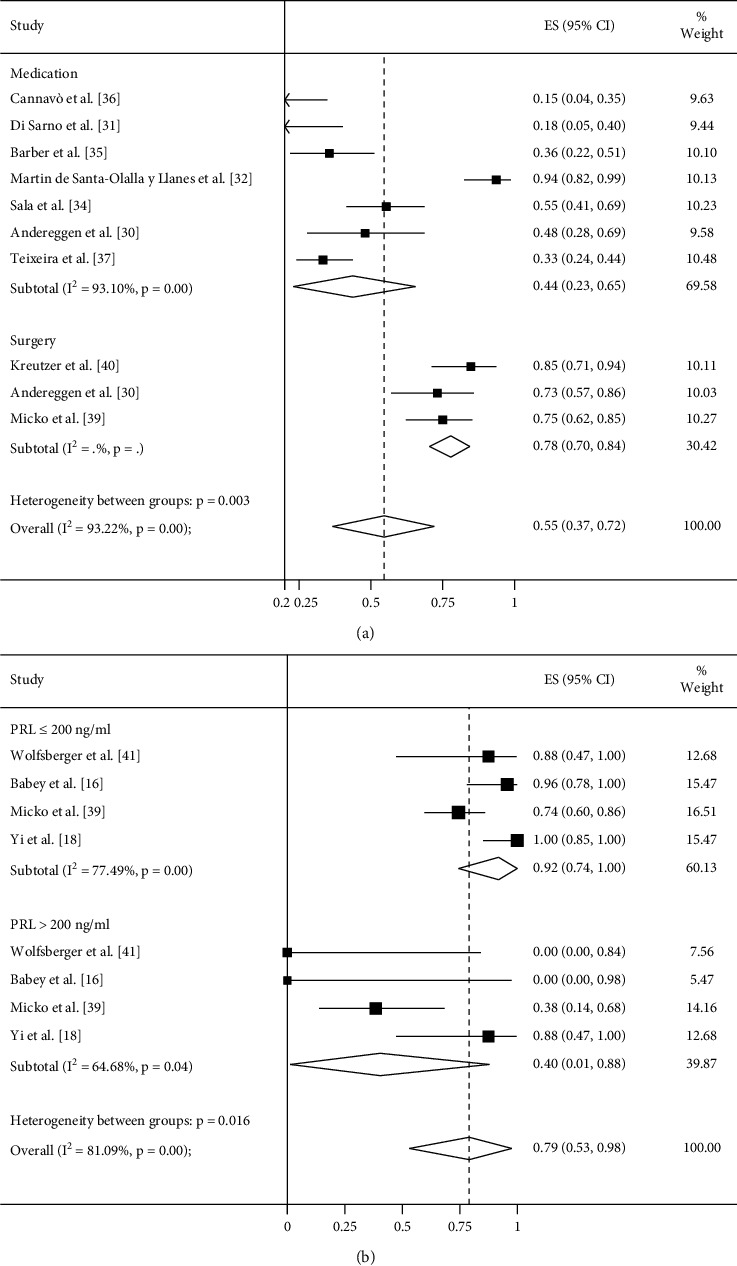
Meta-analysis of remission rates comparing surgery and medical treatment in microprolactinomas after treatment withdrawal and the results of subgroup analysis: (a) the remission rate was achieved in 78% of patients treated with surgery and 44% of patients treated with DAs (*P*=0.003) after treatment withdrawal and (b) the remission rate was achieved in 92% of patients with preoperative PRL level of ≤200 ng/ml and 40% of patients with preoperative PRL level of >200 ng/ml (*P*=0.029).

Meta-analysis results for remission rates of PRL level (reported as a forest plot) are shown in Figures 2–4. There was no significant difference between the surgical treatment and medical treatment groups at ≤3 months of follow-up (89% vs. 78%; *P*=0.092). However, the medical treatment group achieved a higher remission rate at ≥12 months of follow-up (96% vs. 86%; *P*=0.019; Table 2; Figures 2 and 3).

At final follow-up periods, namely, treatment withdrawal (DA withdrawal in the medical group; no other therapy was applied after surgery), the surgery group had an obvious higher remission rate than medical treatment (78% vs. 44%; *P*=0.003; Table 2; Figure 4(a)).

Subgroup analysis of the surgical group showed that patients with preoperative PRL level of ≤200 ng/mL had a significantly higher remission rate than patients with preoperative PRL level of >200 ng/mL (92% vs. 40%; *P*=0.029; Table 2; Figure 4(b)). The pooled complication rates in the surgical group are displayed in Supplemental Table 3.

### 3.2. Risk of Bias

Funnel plots and Egger's test were performed; the results indicated no publication bias after sensitive analysis (supplemental data).

## 4. Discussion

The Endocrine Society's Clinical Practice Guidelines recommend DAs as first-line therapy to lower the PRL level, decrease tumour size, and restore gonadal function for most patients with prolactinoma [8]. However, the high tumour recurrence rate after DA withdrawal remains a clinical problem. Recently, some studies have suggested that surgery could be offered as the initial therapeutic approach for microprolactinomas due to the high remission rate observed after surgery when performed by experienced neurosurgeons [15, 20]. In order to compare the remission rates of initial surgical and medical treatment in treatment-naive microprolactinoma patients, we carried out a systematic review and meta-analysis. Surgery showed a high remission rate after treatment withdrawal (DA withdrawal in the medical group; no other therapy was applied after surgery in the surgical group) in microprolactinomas, particularly in patients with a preoperative PRL level of ≤200 ng/mL.

DAs are highly effective in normalising the PRL level and reducing tumour size and are recommended as the first choice for essentially all patients with prolactinomas. CAB shows greater efficacy and has fewer adverse effects than BRC [43]. TSS is usually reserved as a second-line option for the very small number of patients that do not tolerate or do not respond to DA therapy [8, 44]. In our meta-analysis, the remission rate was relatively high in the initial surgical treatment but low in the initial medical treatment, although there were no significant differences among them (89% vs. 78%) at short-term follow-up (≤3 months). However, the difference emerged at long-term follow-up (≥12 months), where the remission rate of medical treatment was higher. This may be because medical therapy with DA requires to be slowly uptitrated and needs a longer time to take effect. With longer follow-up periods, the remission rate of medication increased in conjunction with treatment duration, while the surgical remission rate remained stable. Nonetheless, the initial surgical treatment achieved a long-term remission rate almost as high as medical treatment (86% vs. 96%).

Although DAs are successfully used in prolactinoma patients, their drawbacks increase over time. The long-term or lifelong requirement of DA therapy and the recurrence risk of hyperprolactinemia after its withdrawal remain critical clinical issues. The Endocrine Society's Clinical Practice Guidelines recommend that DAs may be tapered and perhaps discontinued in patients who have been treated for at least 2 years [8]. However, even after adequate DA treatment for >2 years, high hyperprolactinemia recurrence rates of 79% and 64% after DA withdrawal have been reported by Dekkers et al. [9] and Hu et al. [10], respectively. In a recent meta-analysis with a total of 1,106 patients, the recurrence rate after CAB withdrawal was 63.4% [45]. Our meta-analysis showed a recurrence rate of 56% after DA withdrawal, consistent with previous studies. However, Colao et al. [46] reported a 33% recurrence rate after DA withdrawal in prolactinoma patients, much lower than our and other previous studies; this may due to their stricter inclusion criteria of patients with DA withdrawal. In clinical practice, more patients end up relapsing than remain cured [9, 47].

Recently, there has been an increase in reports regarding the adverse effects of DAs, particularly in patients treated with BRC [48, 49]. Although there was no apparent evidence of cardiac valvular damage found in hyperprolactinemic patients treated with DAs [50], some concern exists regarding the long-term use of small doses of CAB and valvular problems [20]. On the other hand, the development and refinement of endoscopic transsphenoidal surgical techniques over the past 20 years have resulted in increased cure rates and decreased morbidity and mortality. Numerous case series of patients undergoing surgery performed by one or two neurosurgeons in a single centre or by neurosurgeons performing ≥80 pituitary operations per year mostly achieve high remission rates (82%–100%) [14, 16, 19, 40, 42, 51–55]. In our surgery group, the final remission rate was 78%, significantly higher than the medical group remission rate of 44% after DA withdrawal.

Early studies reported a 17% recurrence rate in apparently surgically cured microprolactinoma patients [56]. More recently, however, reported recurrence rates after surgery in microprolactinomas have ranged from 0% to 8.9% [16, 18, 40], which are considerably lower. Therefore, the higher remission rate after surgery in our study after treatment withdrawal may be due to a lower recurrence rate. In addition, some patients who appear to recur after surgery achieve a normal PRL level after 6 or 7 years of follow-up [20]. Meanwhile, the incidence of surgical complications drops with the surgeon's experience. Salvatori reported that the mortality is quite low (0.2%) in experienced centres that perform >25 surgeries a year [20]. Perioperative diabetes insipidus (DI) and electrolyte abnormalities are common; however, permanent DI is rare (<5%) when surgery is performed by experienced neurosurgeons [57]. Other surgical complications such as meningitis, cerebrospinal fluid (CSF) leak, and anterior pituitary deficiency are infrequent [58]. In our study, the only complication was transient DI (3%); permanent DI, CSF leak, meningitis, and mortality did not occur. Our meta-analysis showed that primary surgery may have an advantage over primary medical therapy with respect to final remission in treatment-naive microprolactinoma patients. Surgery has a high remission rate and is feasible as an initial treatment option in addition to DAs.

We additionally found that microprolactinoma patients with a preoperative PRL level of ≤200 ng/mL had a significantly higher rate of biochemical remission than those with a level of >200 ng/mL, in accordance with previous reports [13, 59]. One study noted that approximately 92% of prolactinomas with preoperative PRL level of <100 ng/mL achieved initial remission, compared with 75% of those with preoperative PRL level of 101 ng/mL–200 ng/mL. However, the remission rate was only 37% in the prolactinoma patients with preoperative PRL level of >200 ng/mL [59]. Therefore, our findings support that a preoperative PRL level of <200 ng/mL is a strong predictor of microprolactinoma remission after surgery. In 2014, Salvatori challenged the opinion that “DA therapy fits all prolactinomas” and argued that surgical treatment should be provided as an option for microprolactinomas in an institution with an experienced neurosurgeon [20]. Our study confirms the notion that primary surgery may be a more effective method to achieve final remission in microprolactinoma patients with a preoperative PRL level of ≤200 ng/mL. In addition, it is worth noting that the overall cure rate for initial surgical treatment may be higher without preoperative medical treatment due to DA-induced tumour fibrosis [60]. The role of surgical intervention should be re-evaluated in the management of treatment-naive microprolactinoma patients, particularly those with PRL level of ≤200 ng/mL.

In our study, high heterogeneity was observed among studies included in the surgical treatment group at the short-term follow-up period (*I*^2^ = 73.6), and in the medical treatment group at the long-term follow-up period (*I*^2^ = 74.1) and final follow-up periods, significant heterogeneity was also found between the studies of the medical treatment group (*I*^2^ = 93.1). Firstly, it may be a result of the different definitions of biochemical remission; there were no uniform criteria for remission in these studies. For instance, in the study of Qu et al. [42], remission was defined as postoperative normalisation of a morning basal PRL level of <15 ng/ml; however, the criteria is postoperative basal serum PRL level of <30.74 ng/mL in Yi et al. [18]. Secondly, different approaches of transsphenoidal surgeries were applied (microscope or endoscope), and all the patients had undergone surgery between 1982 and 2017, involving an extended span of time. During this time, tremendous surgical technique advances have been made, especially in the field of endoscope technology. In addition, among the included studies of medical treatment group, there was a lot of difference in used DAs, treatment length, the proportion of gender, time of follow-up, and withdrawal criteria. On the other side, heterogeneity in the included studies of subgroup analysis was significant, *I*^2^ = 77.5 and 64.7, respectively. This might partially be explained by the small number of studies.

The strength and limitations of this study should be noticed. To our knowledge, this is the first meta-analysis that compares the clinical result of DA treatment and surgery in patients with treatment-naive microprolactinoma. Although DAs are recommended as first-line therapy for many years, however, the surgical remission rate is climbing with the development and refinement of TSS. The classical opinion that “DA therapy fits all prolactinomas” is becoming a controversial problem. Furthermore, the limitations of this systematic review and meta-analysis should be considered at the same time. Firstly, no randomised clinical trial was included in the studies, weakening the results. Secondly, the sample size in the medical group was much smaller than the surgery group in the short-term analysis. Finally, only four surgical studies contained data for remission rates stratified by preoperative PRL level.

In conclusion, modern TSS has a high final remission rate in treatment-naive microprolactinoma patients and may be an alternative first-line treatment option in addition to DA, particularly in those with preoperative PRL level of <200 ng/mL. However, the confidence level in the evidence from this meta-analysis is not strongly attributable to the noncomparative nature. Future randomised controlled clinical studies are needed.

## Figures and Tables

**Table 1:** Characteristics of the included studies.

Study identification	Original country of study	Patients (N)	Age (Y; mean)	Female (%)	Intervention patients (N)		Mean follow-up time (months)	Short-term remission rate	Long-term remission rate	Remission rate after treatment withdrawal^a^	Preoperative PRL level, ≤200 ng/mL	Preoperative PRL level of ≤200 ng/mL remission rate	Preoperative PRL level, >200 ng/mL	Preoperative PRL level of >200 ng/mL remission rate
Andereggen et al. [30]	Switzerland	8	NA	0	Medical arm and CAB/BRC	3	63	2 (66.7%)	NA	NA	NA	NA	NA	NA
					Surgical arm and TSS	5		4 (80.0%)	4 (80.0%)	NA	NA	NA	NA	NA

Andereggen et al. [15]	Switzerland	66	NA	100	Medical arm and CAB/BRC	25	90	18 (72.0%)	21 (84.0%)	12 (48.0%)	NA	NA	NA	NA
					Surgical arm and TSS	41		27 (65.9%)	37 (90.2%)	30 (73.2%)	NA	NA	NA	NA

Di Sarno et al. [31]	Italy	22	34.3	95.4	Medication and CAB	22	>24	18 (81.8%)	22 (100%)	4 (18.2%)	12	12 (100%)	10	10 (100%)

Martin de Santa-Olalla y Llanes et al. [32]	Spain	47	29.9	100	Medication and BRC	47	194.4	NA	39 (82.9%)	44 (93.6%)	NA	NA	NA	NA

Vale et al. [33]	USA	35	NA	85.7	Medication and CAB/BRC	35	48	NA	34 (97.1%)	NA	NA	NA	NA	NA

Sala et al. [34]	Italy	56	NA	NA	Medication and CAB	56	>36	NA	56 (100%)	31 (55.3%)	NA	NA	NA	NA
Barber et al. [35]	UK	45	NA	NA	Medication and CAB/BRC	45	49.2	NA	45 (100%)	16 (35.5%)	NA	NA	NA	NA

Cannavò et al. [36]	Italy	26	29.6	84.6	Medication and CAB	26	>36	NA	23 (88.5%)	4 (15.4%)	NA	NA	NA	NA
Teixeira et al. [37]	Portugal	96	NA	NA	Medication and CAB/BRC	96	127.2	NA	NA	32 (33.3%)	NA	NA	NA	NA

Torres-García et al. [38]	Sweden	7	17.4	100	Medication and CAB/BRC	7	120	NA	6 (85.7%)	NA	3	3 (100%)	4	3 (75.0%)

Micko et al. [39]	Austria	60	33.5	83.3	Surgery and TSS	60	37	49 (81.7%)	40 (66.7%)	45 (75.0%)	47	35 (74.5%)	13	5 (38.5%)

Kreutzer et al. [40]	Germany	46	NA	NA	Surgery and TSS	46	19.6	42 (91.3%)	39 (84.8%)	39 (84.8%)	NA	NA	NA	NA
Babey et al. [16]	Switzerland	24	30.2	83.3	Surgery and TSS	24	30.2	22 (91.7%)	22 (91.7%)	NA	23	22 (95.7%)	1	0 (0%)

Yi et al. [18]	China	31	28	100	Surgery and TSS	31	53	30 (96.8%)	28 (90.3%)	NA	23	23 (100%)	8	7 (87.5%)

Tamasauskas et al. [17]	Lithuania	10	31	100	Surgery and TSS	10	50.4	NA	9 (90.0%)	NA	NA	NA	NA	NA

Gnjidic et al. [19]	Croatia	54	NA	NA	Surgery and TSS	54	24	53 (98.1%)	NA	NA	NA	NA	NA	NA

Wolfsberger et al. [41]	Austria	10	NA	0	Surgery and TSS	10	>24	NA	7 (70.0%)	NA	8	7 (87.5%)	2	0 (0%)

Qu et al. [42]	China	18	NA	0	Surgery and TSS	18	45	15 (83.3%)	NA	NA	NA	NA	NA	NA

N, number; Y, year; NA, not available; CAB, cabergoline; BRC, bromocriptine; PRL, prolactin; and TSS, transsphenoidal surgery. ^a^Treatment withdrawal means DA withdrawal in the medical group, and no other therapy was applied after surgery in the surgical group.

**Table 2:** Meta-analysis of remission rates as compared between surgery and medical treatment. Meta-analysis showed that there is no significant difference between the surgery group and medical treatment at short-term follow-up (89% vs. 78%; *P*=0.092). The DA treatment group achieved a higher remission rate at ≥12 months follow-up (96% vs. 86%; *P*=0.019). However, surgery achieved a higher remission rate after treatment withdrawal (78% vs. 44%; *P*=0.003). Subgroup analysis of surgical cohort revealed that patients with preoperative prolactin level of ≤200 ng/mL had a higher remission rate than patients with preoperative prolactin level of >200 ng/mL (92% vs. 40%; *P*=0.029).

Outcomes	Effect size	95% CI	*I*^2^ (%)	Heterogeneity *P*-value	*P*-value (interaction)
Remission FUT ≤ 3 months					
Medical treatment	0.78	(0.64, 0.90)	NA	NA	0.092
Surgery	0.89	(0.79, 0.96)	73.6	0.01	

Remission FUT ≥ 12 months					
Medical treatment	0.96	(0.88, 1.0)	74.4	0.01	0.019
Surgery	0.86	(0.80, 0.91)	7.9	0.37	

Remission after treatment withdrawal^a^					
Medical treatment	0.44	(0.23, 0.65)	93.1	0.01	0.003
Surgery	0.78	(0.70, 0.84)	NA	NA	

Remission surgery					
PRL ≤ 200 ng/mL	0.92	(0.74, 1.0)	77.5	0.01	0.029
PRL > 200 ng/mL	0.40	(0.01, 0.88)	64.7	0.04	

NA, not available; DAs, dopamine agonists; and FUT, follow-up time. ^a^Treatment withdrawal means DA withdrawal in the medical group, and no other therapy was applied after surgery in the surgical group.

## Data Availability

The underlying data supporting the results of this study can be found in the main manuscript and the supplemental files.

## Supplementary Materials

Supplementary Materials Supplemental Table 1: strategy of searches. Supplemental Table 2: assessment of study quality using a modified version of the Newcastle–Ottawa scale for cohort studies. Supplemental Table 3: incidence of surgical complications. Supplemental data: funnel plots and Egger's test.
