# Supplementary material for: Surgery and Medical Treatment in Microprolactinoma: A Systematic Review and Meta-Analysis
Source: Int J Endocrinol. 2021 Aug 30;2021:9930059. doi: 10.1155/2021/9930059 (PMC8423556; doi:10.1155/2021/9930059)
Supplement: Supplementary Materials — Supplemental Table 1: strategy of searches. Supplemental Table 2: assessment of study quality using a modified version of the Newcastle–Ottawa scale for cohort studies. Supplemental Table 3: incidence of surgical complications. Supplemental data: funnel plots and Egger's test. [file 9930059.f1.zip › 9930059.f1/Supplemental data.docx]

**Funnel plots and Egger’s test**

1. Funnel plots and Egger’s test between studies of medication on the short-term follow up (≤3 months)

Appendix 1. (a-b) Sensitivity analysis and Egger’s test showed no publication bias (P=0.659) between studies of medication on the Short-term Remission Rate.

Fig 1. (c) Funnel plot of medication groups on the Short-term Remission Rate.

2. Funnel plots and Egger’s test between studies of surgery on the short-term follow up (≤3 months)

Appendix 2. (a-b) Sensitivity analysis and Egger’s test showed no publication bias (P=0.837) between studies of surgery on the Short-term Remission Rate.

Fig 2. (c) Funnel plot of surgery groups on the Short-term Remission Rate.

3. Funnel plots and Egger’s test between studies of medication on the long-term follow up (≥12 months)

Appendix 3. (a-b) Sensitivity analysis and Egger’s test showed no publication bias (P=0.354) between studies of medication on the Long-term Remission Rate.

Fig 3. (c) Funnel plot of medication groups on the Long-term Remission Rate.

4. Funnel plots and Egger’s test between studies of surgery on the long-term follow up (≥12 months)

Appendix 4. (a-b) Sensitivity analysis and Egger’s test showed no publication bias (P=0.956) between studies of surgery on the Long-term Remission Rate.

Fig 4. (c) Funnel plot of surgery groups on the Long-term Remission Rate.

5. Funnel plots and Egger’s test between studies of medication after treatment withdrawal

Appendix 5. (a-b) Sensitivity analysis showed a study (by Martín et al.) led to unstable outcome and Egger’s test showed no publication bias (P=0.795) between studies of medication on the Remission Rate without DAs.

Fig 5. (c) Funnel plot of medication groups on the Remission Rate without DAs.

6. Funnel plots and Egger’s test between studies of surgery after treatment withdrawal

Appendix 6. (a-b) Sensitivity analysis and Egger’s test showed no publication bias (P=0.340) between studies of surgery on Remission Rate without DAs.

Fig 6. (c) Funnel plot of surgery groups on Remission Rate without DAs.

7. Funnel plots and Egger’s test between studies of surgery for patients with PRL≤200 ng/ml

Appendix 7. (a-b) Sensitivity analysis showed a study (Micko et al.) made unstable outcome and Egger’s test showed no publication bias (P=0.561) between studies of surgery on the Remission Rate for patients with PRL≤200 ng/ml.

Fig 7. (c) Funnel plot of surgery groups on the Remission Rate for patients with PRL≤200 ng/ml.

8. Funnel plots and Egger’s test between studies of surgery for patients with PRL＞200 ng/ml

Appendix 8. (a-b) Sensitivity analysis and Egger’s test showed no publication bias (P=0.514) between studies of surgery on the Remission Rate for patients with PRL＞200 ng/ml.

Fig 8. (c) Funnel plot of surgery groups on the Remission Rate for patients with PRL＞200 ng/ml.
