# Supplementary material for: Surgery and Medical Treatment in Microprolactinoma: A Systematic Review and Meta-Analysis
Source: Int J Endocrinol. 2021 Aug 30;2021:9930059. doi: 10.1155/2021/9930059 (PMC8423556; doi:10.1155/2021/9930059)
Supplement: Supplementary Materials — Supplemental Table 1: strategy of searches. Supplemental Table 2: assessment of study quality using a modified version of the Newcastle–Ottawa scale for cohort studies. Supplemental Table 3: incidence of surgical complications. Supplemental data: funnel plots and Egger's test. [file 9930059.f1.zip › 9930059.f1/supplemental table 1.docx]

| **Supplemental Table 1.** Searches Strategy | | |  |
| --- | --- | --- | --- |
| No. | Query | Results |  |
| #1 | 'prolactinoma'/exp | 5822 |  |
| #2 | 'prolactinomas' OR 'lactotroph adenoma' OR 'adenoma, lactotroph' OR 'adenomas, lactotroph' OR 'lactotroph adenomas' OR 'prolactinoma, familial'OR 'prl-secreting pituitary adenoma' OR 'prl secreting pituitary adenoma' OR 'prl-secreting pituitary adenomas' OR 'pituitary adenoma, prl-secreting'OR 'pituitary adenomas, prl-secreting' OR 'prolactin-producing pituitary adenoma' OR 'pituitary adenoma, prolactin-producing' OR 'pituitary adenomas, prolactin-producing' OR 'prolactin producing pituitary adenoma' OR 'prolactin-producing pituitary adenomas' OR 'prolactin-secreting pituitary adenoma' OR 'prolactin secreting pituitary adenoma' OR 'adenoma, prolactin-secreting, pituitary' OR 'pituitary adenoma, prolactin-secreting'OR 'pituitary adenoma, prolactin secreting' OR 'pituitary adenomas, prolactin-secreting' OR 'prolactin-secreting pituitary adenomas' OR 'microprolactinoma' OR 'microprolactinomas' OR 'macroprolactinoma' OR 'macroprolactinomas':ab,ti | 3482 |  |
| #3 | #1 OR #2 | 6597 |  |
| #4 | 'drug therapy'/exp | 2465841 |  |
| #5 | 'dopamine receptor stimulating agent'/exp | 199997 |  |
| #6 | 'bromocriptine'/exp | 20237 |  |
| #7 | 'cabergoline'/exp | 5321 |  |
| #8 | 'drug' OR 'therapy, drug' OR 'drug therapies' OR 'therapies, drug' OR 'chemotherapy chemotherapies' OR 'pharmacotherapy' OR 'pharmacotherapies' OR 'agonists, dopamine' OR 'receptor agonists, dopamine' OR 'dopamine receptor agonist' OR 'agonist, dopamine receptor' OR 'receptor agonist, dopamine' OR 'dopamine receptor agonists' OR 'dopaminergic agonists' OR 'agonists, dopaminergic' OR 'dopaminergic agonist'OR 'agonist, dopaminergic' OR 'agonists, dopamine receptor' OR 'dopamine agonist' OR 'agonist, dopamine' OR '2-bromo-alpha-ergokryptine' OR '2 bromo alpha ergokryptine' OR 'bromocryptin' OR '2-bromoergokryptine' OR '2 bromoergokryptine' OR 'bromocriptin' OR '2-bromoergocryptine' OR '2 bromoergocryptine' OR '2-bromo-alpha-ergocryptine' OR '2 bromo alpha ergocryptine' OR 'cb-154' OR 'cb 154' OR 'cb154' OR 'parlodel' OR '2-bromoergocryptine mesylate' OR '2 bromoergocryptine mesylate' OR 'mesylate, 2-bromoergocryptine' OR 'bromocriptine mesylate' OR 'mesylate, bromocriptine' OR '2-bromoergocryptine methanesulfonate' OR '2 bromoergocryptine methanesulfonate' OR 'methanesulfonate, 2-bromoergocryptine' OR '1-((6-allylergolin-8beta-yl)carbonyl)-1-(3-(dimethylamino)propyl)-3-ethylurea' OR 'galastop' OR 'fce 21336' OR 'fce-21336'OR 'cabaser' OR 'cabaseril' OR 'dostinex' OR 'cabergoline diphosphate':ab,ti | 10697224 | |
| #9 | #5 OR #6 OR #7 OR #8 OR #9 | 11103713 |  |
| #10 | 'surgery'/exp | 4542603 |  |
| #11 | 'endoscopy'/exp | 568825 |  |
| #12 | 'microscopy'/exp | 790264 |  |
| #13 | operative therapy' OR 'operative procedures' OR 'invasive procedures' OR 'operations' OR 'peroperative procedures' OR 'perioperative procedures'OR 'preoperative procedures' OR 'intraoperative procedures' OR 'surgical procedures, endoscopic' OR 'procedure, endoscopic surgical' OR 'procedures, endoscopic surgical' OR 'surgical procedure, endoscopic' OR 'endoscopy, surgical' OR 'surgical endoscopy' OR 'endoscopic surgical procedure' OR 'endoscopic surgical procedures' OR 'optical microscopy' OR 'microscopy, optical' OR 'simple microscopy' OR 'microscopy, simple'OR 'light microscopy' OR 'microscopy, light' OR 'compound microscopy' OR 'microscopy, compound' OR 'hand-held microscopy' OR 'hand held microscopy' OR 'microscopy, hand-held' OR 'transphenoidal':ab,ti | 261281 |  |
| #14 | #11 OR #12 OR #13 OR #17 | 5579814 |  |
| #15 | #3 AND #10 AND #18 | 1448 |  |
| Embase 1970-2020 Nov | |  |  |
