# Supplementary material for: Surgery and Medical Treatment in Microprolactinoma: A Systematic Review and Meta-Analysis
Source: Int J Endocrinol. 2021 Aug 30;2021:9930059. doi: 10.1155/2021/9930059 (PMC8423556; doi:10.1155/2021/9930059)
Supplement: Supplementary Materials — Supplemental Table 1: strategy of searches. Supplemental Table 2: assessment of study quality using a modified version of the Newcastle–Ottawa scale for cohort studies. Supplemental Table 3: incidence of surgical complications. Supplemental data: funnel plots and Egger's test. [file 9930059.f1.zip › 9930059.f1/supplemental table 2.docx]

| **Supplemental table 2:** Assessment of study quality using a modified version of the Newcastle-Ottawa scale for cohort studies | | | | | | |
| --- | --- | --- | --- | --- | --- | --- |
| Study | Study population cleared defined | Consecutive patients included | Assessment of outcome well performed | Follow up long enough for outcomes to occur(>1.5y) | Surgical technical well defined | Adequacy of follow up of cohort |
| Andereggen, 2017 | 1 | 1 | 1 | 1 | 1 | 1 |
| Andereggen, 2017 | 1 | 1 | 1 | 1 | 1 | 1 |
| Di Sarno, 2000 | 1 | 1 | 1 | 1 | NA | 1 |
| Martin, 2013 | 1 | 1 | 1 | 1 | NA | 1 |
| Vale, 2013 | 1 | 1 | 1 | 1 | NA | 1 |
| Sala, 2016 | 1 | 1 | 1 | 1 | NA | 1 |
| Barber, 2011 | 1 | 1 | 1 | 1 | NA | 1 |
| Cannavo, 1999 | 1 | 1 | 1 | 1 | NA | 1 |
| Teixeira, 2017 | 1 | 1 | 1 | 1 | NA | 1 |
| Torres-Garcia, 2018 | 1 | 1 | 1 | 1 | NA | 1 |
| Micko, 2018 | 1 | 1 | 1 | 1 | 1 | 1 |
| Kreutzer, 2008 | 1 | 1 | 1 | 1 | 1 | 1 |
| Babey, 2011 | 1 | 1 | 1 | 1 | 1 | 1 |
| Yi, 2018 | 1 | 1 | 1 | 1 | 1 | 1 |
| Tamasauskas, 2012 | 1 | 1 | 1 | 1 | 1 | 1 |
| Gnjidic, 2014 | 1 | 1 | 1 | 1 | 1 | 1 |
| Wolfsberger, 2003 | 1 | 1 | 1 | 1 | 1 | 1 |
| Qu, 2011 | 1 | 1 | 1 | 1 | 1 | 1 |
| Abbreviations: NA, not available; 1=yes; 0=no | | | | | | |
