# Supplementary material for: Surgery and Medical Treatment in Microprolactinoma: A Systematic Review and Meta-Analysis
Source: Int J Endocrinol. 2021 Aug 30;2021:9930059. doi: 10.1155/2021/9930059 (PMC8423556; doi:10.1155/2021/9930059)
Supplement: Supplementary Materials — Supplemental Table 1: strategy of searches. Supplemental Table 2: assessment of study quality using a modified version of the Newcastle–Ottawa scale for cohort studies. Supplemental Table 3: incidence of surgical complications. Supplemental data: funnel plots and Egger's test. [file 9930059.f1.zip › 9930059.f1/supplemental table 3 (1).docx]

| **Supplemental Table 3.** The incidence of surgical complications | | | | | |
| --- | --- | --- | --- | --- | --- |
| **Complications** |  | **Effect Size** | **95% CI** | **I^2^, %** | **Heterogeneity *P* Value** |
| Transitory diabetes |  | 0.03 | (0.00, 0.10) | 83.9 | 0.01 |
| Permanent diabetes |  | 0.00 | (0.00, 0.01) | 30.9 | 0.2 |
| Transient syndrome of inappropriate secretion of ADH | | 0.01 | (0.00, 0.04) | 56.8 | 0.01 |
| CSF leakage |  | 0.00 | (0.00, 0.00) | 0.0 | 0.61 |
| Meningitis |  | 0.00 | (0.00, 0.00) | 0.0 | 1.0 |
| Overall |  | 0.16 | (0.07, 0.27) | 83.4 | 0.01 |
| Long overall |  | 0.03 | (0.00, 0.08) | 69.1 | 0.01 |

Abbreviations: ADH, antidiuretic hormone; CSF, cerebrospinal fluid
